# Are There Sex Differences in Brain Activity in Response to Active Tactile Stimulation?

**DOI:** 10.7759/cureus.92170

**Published:** 2025-09-12

**Authors:** Kei Sasaki, Nobukiyo Yoshida, Misuzu Oishi, Ryo Kawamura, Naoki Kodama

**Affiliations:** 1 Department of Radiological Technology, Faculty of Medical Technology, Niigata University of Health and Welfare, Niigata, JPN; 2 Graduate School of Health and Welfare, Niigata University of Health and Welfare, Niigata, JPN

**Keywords:** active tactile stimulation, fmri brain activation, hardness levels, postcentral gyrus, sensorimotor processing, sex differences, tactile perception

## Abstract

Tactile information plays a crucial role in object recognition, spatial localization, and motor control. In elucidating the neural basis of these processes, it is critically important to clarify the underlying neural mechanisms of active tactile stimulation. This study aimed to examine brain activation patterns elicited by active tactile stimulation using stress balls of different hardness levels (soft, medium, and hard) with functional magnetic resonance imaging (fMRI) and to investigate their relationship with sex differences and subjective evaluations. Participants were 77 healthy right-handed young adults (37 males and 40 females) who performed a task involving gripping a stress ball at a force of 5 kg once per second under each condition. Following the scanning session, subjective ratings of hardness and comfort were obtained using a nine-point Likert scale. Across all conditions, activation was consistently observed in sensorimotor-related regions such as the postcentral gyrus, precentral gyrus, thalamus, and cerebellum, indicating stable engagement of these areas during active tactile processing. Notably, the medium condition elicited additional activation in higher-order cognitive and emotional regions, including the angular gyrus, posterior cingulate cortex, and hippocampus, suggesting that moderate stimulation may enhance introspective processing and emotional evaluation. Although no statistically significant differences were detected between sexes in the contrast analyses, subjective evaluations showed that, in the medium condition only, males rated the stimulation as significantly more pleasant than females. These findings indicate that active tactile stimulation primarily engages the sensorimotor network, while the strength and perceived comfort of the stimulus can influence emotional and cognitive processing. Furthermore, although clear sex-related differences in neural activity were not observed, perceptual differences between males and females may still occur.

## Introduction

Touch is one of the most fundamental senses in the somatosensory system, playing a crucial role in object recognition and motor control in daily life. Neural responses to tactile stimulation are primarily processed in the primary (S1) and secondary (S2) somatosensory cortices, with distinct activation patterns emerging depending on the type and intensity of stimulation [1,2]. Tactile stimulation can be broadly categorized into passive and active forms, which are mediated by different neural mechanisms. Passive stimulation involves the application of external stimuli to the skin and primarily engages S1, the insular cortex, and the supplementary motor area (SMA), depending on stimulus intensity and location [1]. In contrast, active stimulation involves self-initiated exploration of tactile stimuli, recruiting not only sensory processing areas but also regions associated with motor planning and control. Active stimulation has been shown to engage S1, S2, the premotor cortex (PMC), SMA, and the cerebellum [3,4].

Johansson and Flanagan demonstrated that during active exploratory behavior, the recognition of surface texture and hardness involves not only somatosensory cortices but also motor-related areas [5]. Moreover, cortical activity in tactile processing regions increases with greater expertise in task performance [1]. Neural responses to active tactile stimulation, therefore, reflect the integration of sensory input, motor control, and sensorimotor coordination. Emotional evaluation of pleasantness or unpleasantness activates the insula and amygdala, whereas motor feedback and motor learning recruit the cerebellum [6]. Pleger et al. further reported that active tactile stimulation engages more widespread brain areas than passive stimulation, with particular emphasis on SMA and PMC involvement [1].

The integration of tactile and visual information, referred to as visuotactile integration, plays a critical role in object recognition, spatial localization, and motor control. Although tactile and visual modalities are distinct, they are integrated within the brain to support more accurate and adaptive behavior. This integration occurs in the intraparietal sulcus (IPS) [7], temporoparietal junction (TPJ) [8], insula [9], S1, and visual cortex (V1) [10-12].

Sex differences in tactile sensitivity and brain activation patterns have been reported [13]. Differences have been observed in responses to painful and pleasant stimuli, as well as in tactile detection thresholds [14]. In general, women are considered to have greater tactile sensitivity than men and can detect weaker stimuli, particularly pleasant ones [13]. For example, women are more likely than men to appreciate non-sexual touch from unfamiliar individuals [14]. In contrast, men tend to interpret touch from peers of equal status as a dominant gesture, whereas women are more likely to interpret it as warm and friendly [15]. Studies using passive tactile stimulation have also shown that women exhibit different emotional responses from men when touched with various textures on different body sites. While both sexes generally rate smooth or soft materials as pleasant, especially on the hand, forearm, or thigh, sex differences emerge for rough textures: men find denim or terry cloth on the forehead unpleasant, whereas women rate these textures on the thigh as most unpleasant [16].

Despite these reports of sex differences in tactile perception, the neural mechanisms underlying active tactile stimulation remain unclear. Among neuroimaging techniques, such as magnetic resonance imaging (MRI), positron emission tomography (PET), electroencephalography (EEG), and magnetoencephalography (MEG), MRI provides a noninvasive approach with high spatial resolution, enabling simultaneous assessment of brain anatomy and function, making it particularly well-suited for studying brain responses to tactile stimulation. We have previously used functional MRI (fMRI) to identify brain regions activated by active tactile stimulation and to clarify differences in brain responses to various textures and material properties [6,17]. However, to our knowledge, no study has examined sex differences in brain activation patterns in response to active tactile stimulation using fMRI. While numerous studies have addressed passive stimulation, research on active tactile stimulation and its associated sex differences remains limited. Therefore, the present study aimed to identify brain activation patterns during active tactile stimulation using fMRI, with a specific focus on clarifying sex-related differences in neural activity.

## Materials and methods

Participants

Seventy-seven healthy adults aged 20 years or older (40 women, 37 men; mean age ± SD: 21.9 ± 1.6 years) participated in the study. All participants were right-handed and had no history of psychiatric disorders or current use of psychoactive medication. The study was approved by the Research Ethics Committee of Niigata University of Health and Welfare (approval number: 18992-230203). All procedures conformed to the principles of the Declaration of Helsinki. Written informed consent was obtained from all participants, and each completed a screening questionnaire to confirm MRI safety before scanning.

Stimuli task

**Figure 1:**
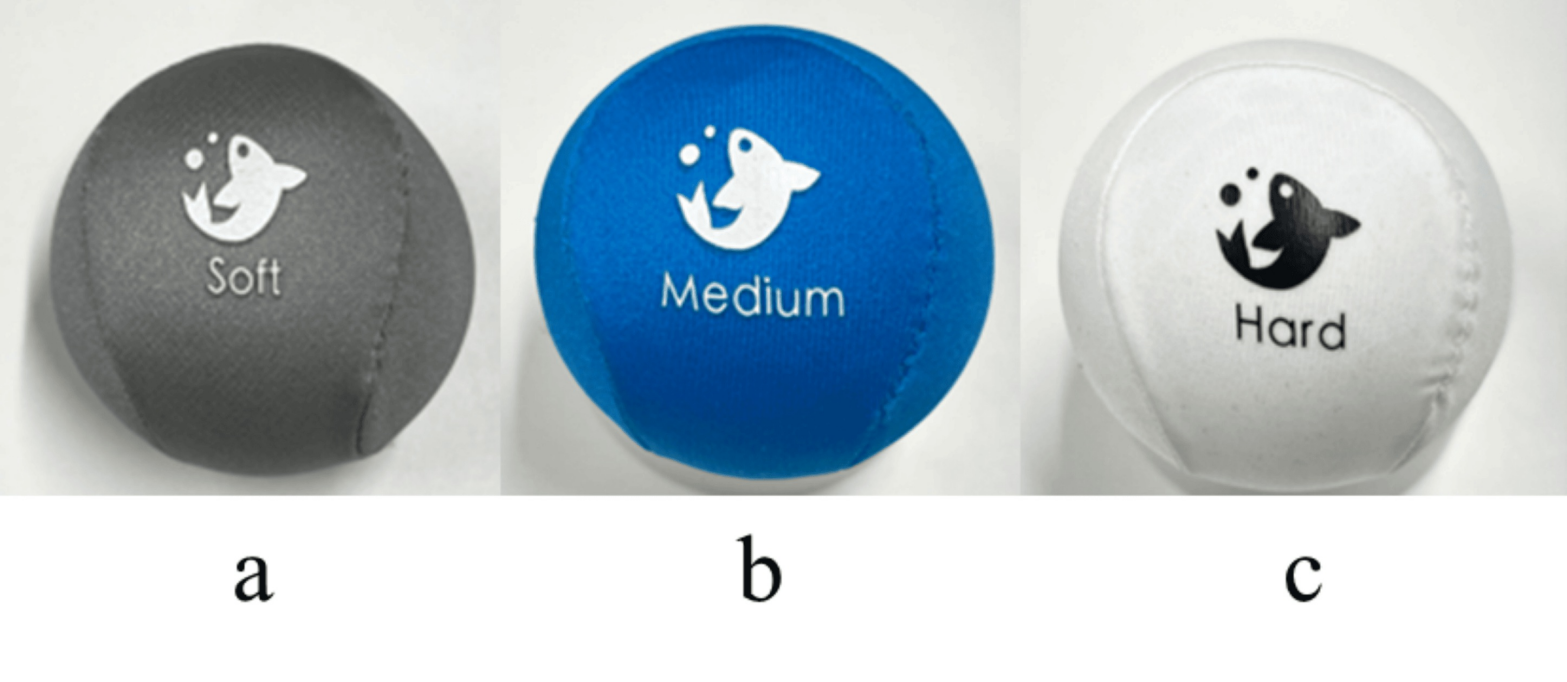
Stress balls used in the experiment a: soft, b: medium, c: hard.

The stress balls used in this study (Serenilite, Great Neck, NY, USA) are shown in Figure 1. Three hardness levels were employed: soft, medium, and hard. Each stress ball consisted of a smooth Lycra outer surface and an internal core made of high-resistance gel. The hardness of each ball was measured using a Qiilu digital durometer (Type A), yielding values of 0.5 HA for soft, 2.5 HA for medium, and 5.5 HA for hard. During the task, participants were instructed to grip the stress ball at a pace of once per second with a force of 5 kg. Before entering the MRI room, participants practiced this grip force using a handgrip dynamometer to become familiar with the target force, and a metronome was used to maintain the one-second gripping pace. To eliminate visual bias, participants were not shown the stress balls before the experiment, and all scanning was conducted with their eyes closed.

Block design

**Figure 2:**
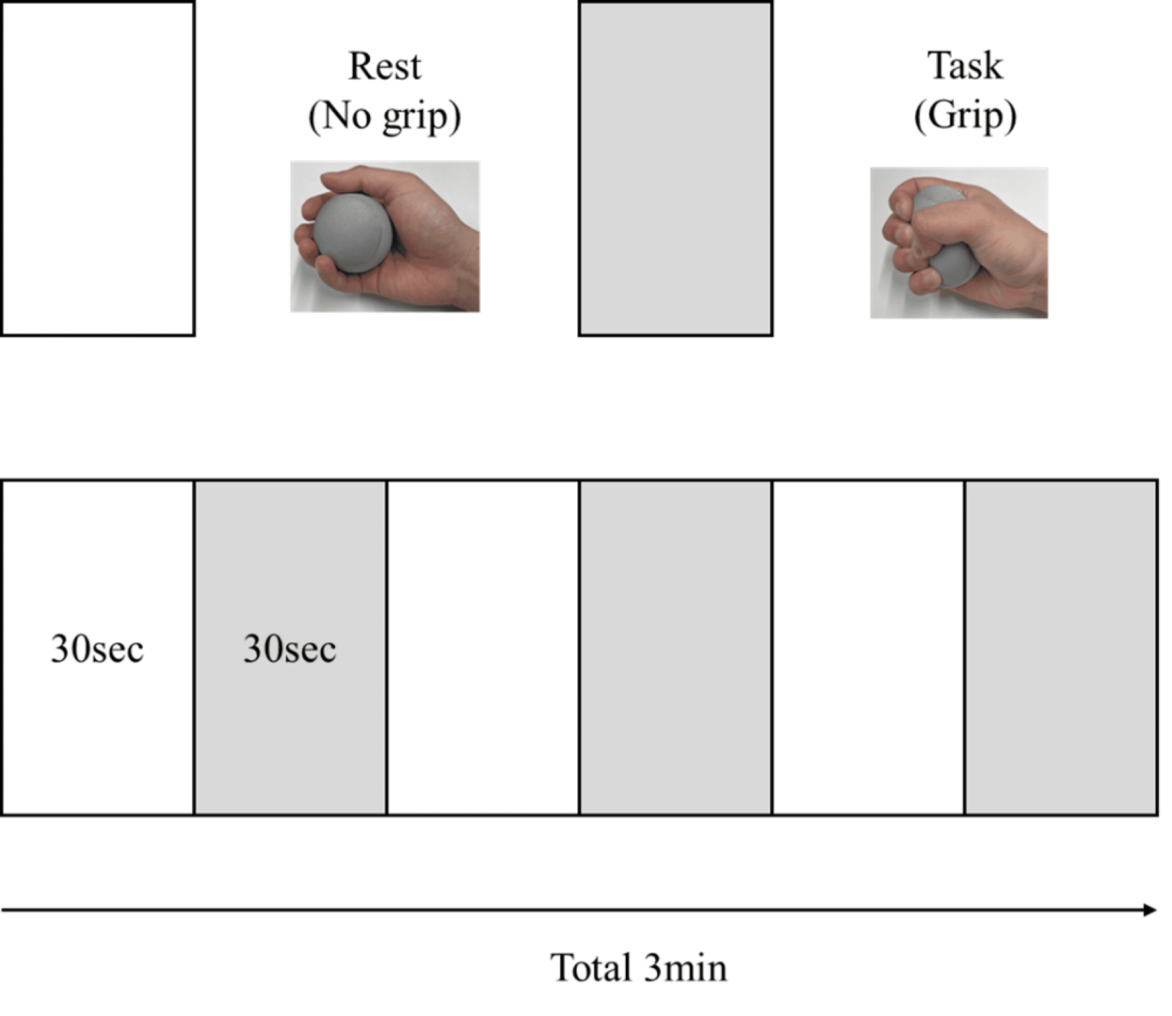
Block design The Rest (No grip) condition lasted for 30 seconds, followed by the Task (Grip) condition for 30 s, repeated three times, for a total duration of 3 min

The block design used in this study is shown in Figure 2 [6]. In the Rest (No grip) condition, participants held the stress ball without movement. In the Task (Grip) condition, participants squeezed the stress ball at a force of 5 kg once per second. Each block consisted of 30 seconds of rest followed by 30 seconds of task, repeated three times for a total duration of 3 minutes. As three types of stress balls were used, the block sequence was repeated three times, with a different stress ball used for each run.

Apparatus

Imaging was performed using a 3-Tesla MRI scanner equipped with a 32-channel head coil (Vantage Galan, Canon Medical Systems, Tochigi, Japan). Participants lay supine on the scanner bed with their arms extended alongside their bodies and palms facing upward. To minimize head motion during ball gripping, foam pads were positioned between the head and the head coil for stabilization. The stress ball was placed in the participant’s right palm, and after each block, it was replaced by the experimenter. To control for order effects, the sequence of the three stress ball hardness conditions was randomized across participants.

MRI acquisition

High-resolution anatomical images were obtained before fMRI acquisition using a T1-weighted magnetization-prepared rapid-gradient-echo (MP-RAGE) sequence. Acquisition parameters were: repetition time (TR) = 5.8 ms, echo time (TE) = 2.7 ms, inversion time (TI) = 900 ms, flip angle (FA) = 9°, matrix = 256 × 256, field of view (FOV) = 230 × 230 mm, and slice thickness = 1.2 mm. Functional images were acquired using an echo-planar imaging sequence with the following parameters: TR = 2,000 ms, TE = 25 ms, FA = 85°, matrix = 64 × 64, FOV = 240 × 240 mm, and slice thickness = 3 mm, covering the entire brain.

fMRI data analyses

The fMRI data were preprocessed and analyzed using Statistical Parametric Mapping 12 (SPM12; Wellcome Trust Center for Neuroimaging, London, UK) implemented in MATLAB (MathWorks Inc., Natick, MA, USA). Slice-timing correction was applied to account for temporal differences in slice acquisition. Motion correction was performed by realignment using estimated motion parameters. Functional images were co-registered to the corresponding high-resolution structural images to correct for spatial discrepancies, followed by normalization of each participant’s brain to the Montreal Neurological Institute (MNI) template space. The normalized images were spatially smoothed using an 8-mm full-width at half-maximum Gaussian kernel.

Following preprocessing, a general linear model was applied within a block design framework to examine brain activity associated with active touch stimulation for each condition. At the first level (individual analysis), contrast images were generated separately for men and women for the following conditions: (1) soft = 1, medium = 0, hard = 0; (2) soft = 0, medium = 1, hard = 0; and (3) soft = 0, medium = 0, hard = 1. At the second level (group analysis), independent two-sample t-tests were performed using the first-level contrast images for each sex. The initial voxel-level threshold was set at an uncorrected p < 0.001, and results were considered significant if they survived family-wise error (FWE) correction at p < 0.05 at the peak level.

Questionnaire

After the fMRI session, participants completed a questionnaire based on a nine-point Likert scale to assess their perceptual experience of each stress ball. Two attributes were rated for each of the three stress ball conditions (soft, medium, hard): softness/hardness and pleasantness/unpleasantness. Statistical analyses were performed using SPSS Statistics 27.0 (IBM Corp., Armonk, NY, USA). For both attributes, sex differences were evaluated using the Mann-Whitney U test. The significance threshold was set at p < 0.05.

## Results

We identified brain activation patterns associated with gripping each of the three stress ball conditions (soft, medium, and hard) in the full participant group, as well as separately for male and female participants. In addition, differential contrast analyses between sexes were conducted to examine potential sex-related differences in brain activation patterns.

Overall brain activation

**Figure 3:**
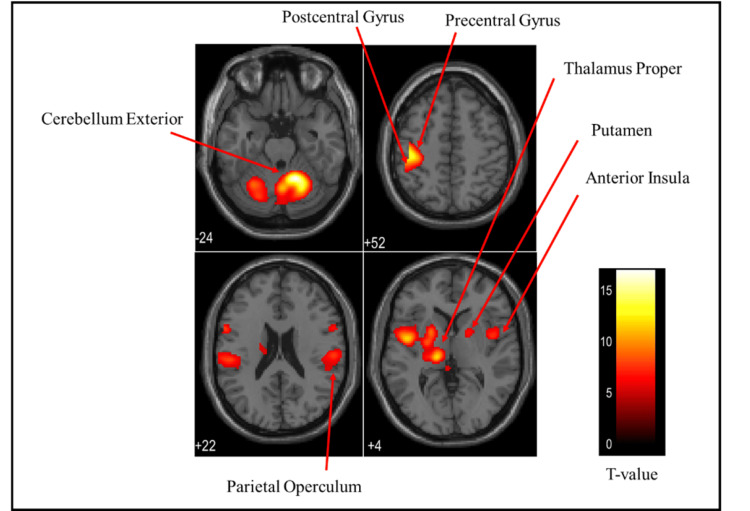
Brain activation regions in all participants (both males and females) during gripping of the soft stress ball The color bar represents T-values in the activation maps

**Figure 4:**
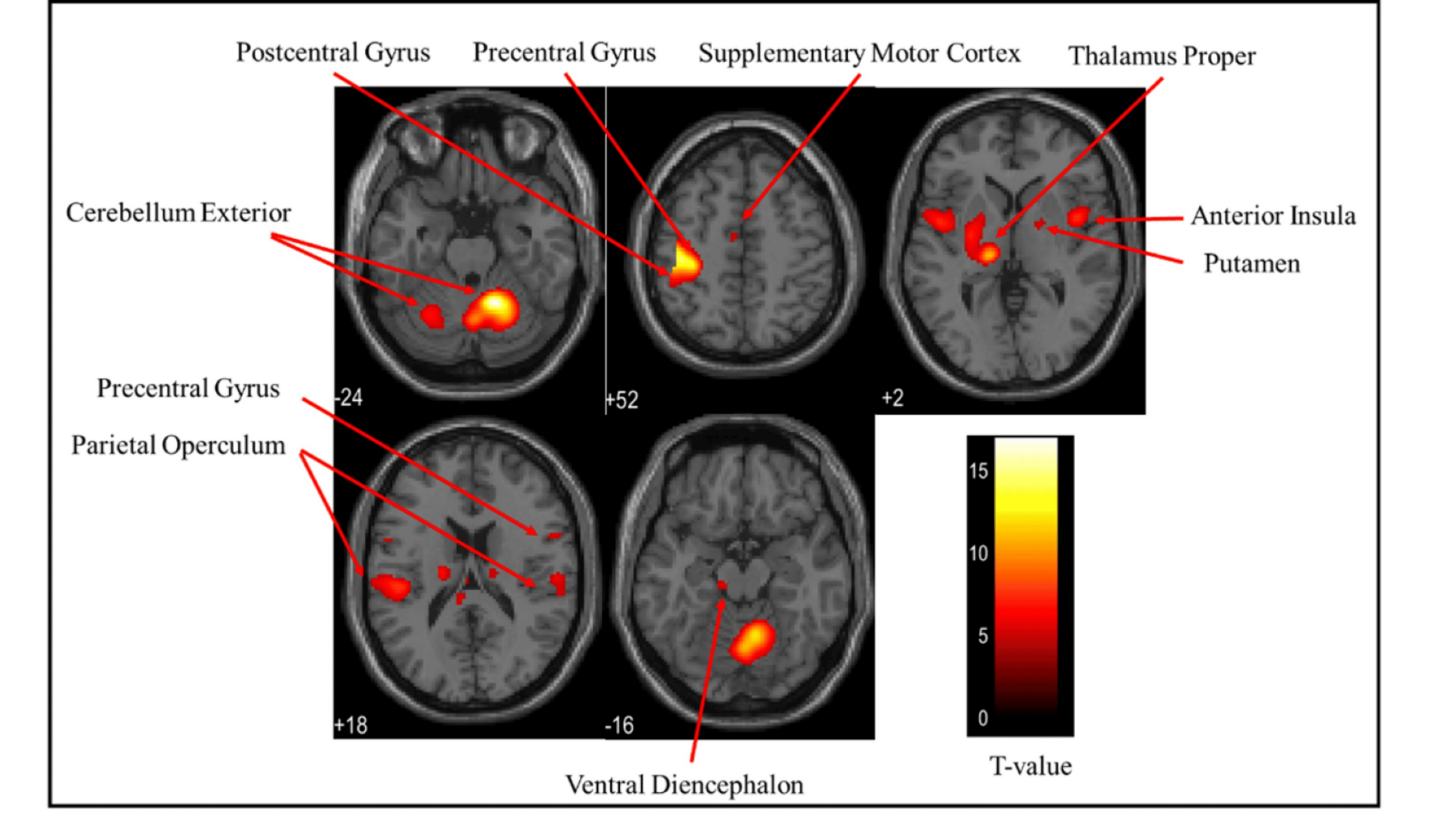
Brain activation regions in all participants (both males and females) during gripping of the medium stress ball The color bar represents T-values in the activation maps

**Figure 5:**
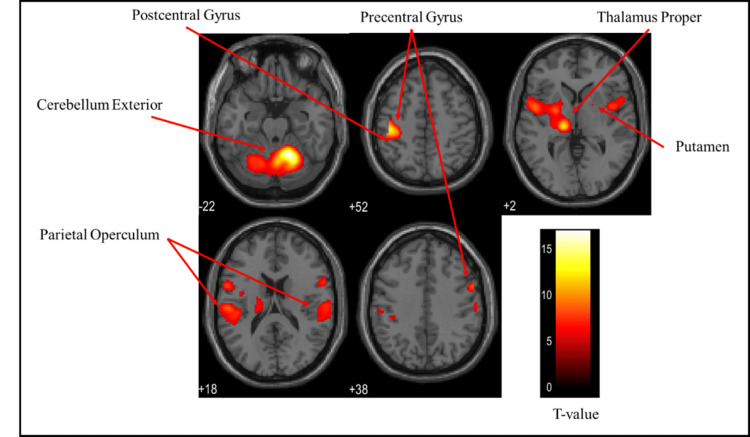
Brain activation regions in all participants (both males and females) during gripping of the hard stress ball The color bar represents T-values in the activation maps

Figures 3-5 and Table 1 present the brain regions activated in all participants (both males and females) while gripping each type of stress ball (soft, medium, hard). Figure 3 shows the activation areas during gripping of the soft stress ball, with significant activation in the cerebellum exterior, postcentral gyrus, precentral gyrus, thalamus proper, parietal operculum, anterior insula, and pallidum. Figure 4 illustrates activation areas during gripping of the medium stress ball, revealing significant activation in the cerebellum exterior, postcentral gyrus, precentral gyrus, thalamus proper, parietal operculum, anterior insula, posterior cingulate gyrus, putamen, hippocampus, supplementary motor cortex, angular gyrus, and supramarginal gyrus. Figure 5 depicts activation areas during gripping of the hard stress ball, with significant activation in the cerebellum exterior, postcentral gyrus, thalamus proper, precentral gyrus, parietal operculum, and putamen.

**Table 1:** Brain activation regions and corresponding Montreal Neurological Institute (MNI) coordinates during gripping of each stress ball in all participants (both males and females) * One-sample t-test FWE: family-wise error

	Hemisphere	Locations	Cluster p－value (FWE)	Cluster size (voxels)	T－ value	X [mm]	Y [mm]	Z [mm]
Soft*	Right	Cerebellum Exterior	<0.001	3316	15.75	18	-54	-24
	Left	Postcentral Gyrus	<0.001	1526	10.47	-48	-24	48
	Left	Precentral Gyrus	<0.001	1526	12.47	-42	-26	52
	Left	Thalamus Proper	<0.001	3033	11.03	-14	-22	4
	Right	Parietal Operculum	<0.001	669	7.21	58	-20	22
	Right	Anterior insula	<0.001	537	7.18	44	2	2
	Right	Pallidum	<0.001	99	6.37	20	4	2
Medium*	Right	Cerebellum Exterior	<0.001	1837	16.98	16	-54	-24
	Left	Postcentral Gyrus	<0.001	845	14.36	-38	-32	54
	Left	Precentral Gyrus	<0.001	845	14.36	-37	-26	54
	Left	Thalamus Proper	<0.001	1608	11.03	-14	-22	2
	Left	Cerebellum Exterior	<0.001	303	8.58	-28	-60	-30
	Left	Parietal Operculum	<0.001	290	7.60	-48	-26	18
	Right	Anterior insula	<0.001	247	7.38	44	4	2
	Left	Posterior Cingulate Gyrus	<0.001	113	6.96	-4	-34	12
	Right	Parietal Operculum	0.001	66	5.87	58	-20	18
	Right	Putamen	0.002	64	5.83	22	0	-4
	Right	Thalamus Proper	0.003	29	5.66	16	-14	20
	Left	Hippocampus	0.004	23	5.58	-12	-24	-16
	Right	Precentral Gyrus	0.006	34	5.46	54	8	16
	Left	Supplementary Motor Cortex	0.017	9	5.15	-6	-10	52
	Right	Angular Gyrus	0.046	2	4.84	50	-48	50
	Right	Supramarginal Gyrus	0.048	1	4.85	60	-40	42
Hard*	Right	Cerebellum Exterior	<0.001	3930	16.40	18	-56	-22
	Left	Postcentral Gyrus	<0.001	765	12.05	-40	-26	52
	Left	Thalamus Proper	<0.001	3120	11.82	-14	-22	2
	Left	Precentral Gyrus	<0.001	3120	9.70	-48	2	8
	Left	Parietal Operculum	<0.001	684	8.25	-54	-20	18
	Right	Precentral Gyrus	<0.001	892	9.19	56	6	38
	Right	Parietal Operculum	<0.001	746	7.13	60	-20	20
	Right	Putamen	0.012	20	5.25	22	4	-2

Brain activation regions in the female group

**Figure 6:**
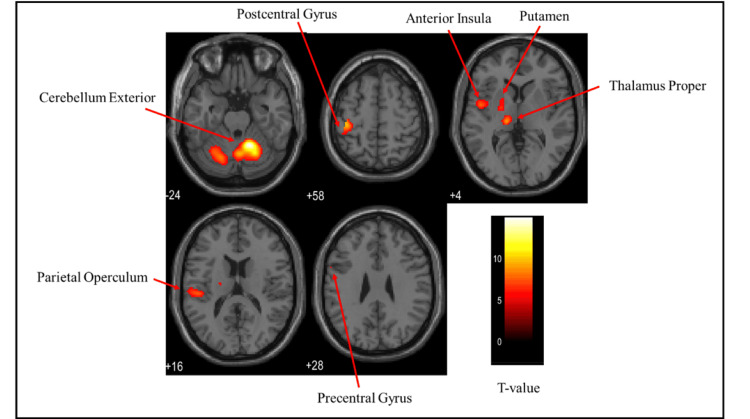
Brain activation regions in the female group during gripping of the soft stress ball The color bar represents T-values in the activation maps

**Figure 7:**
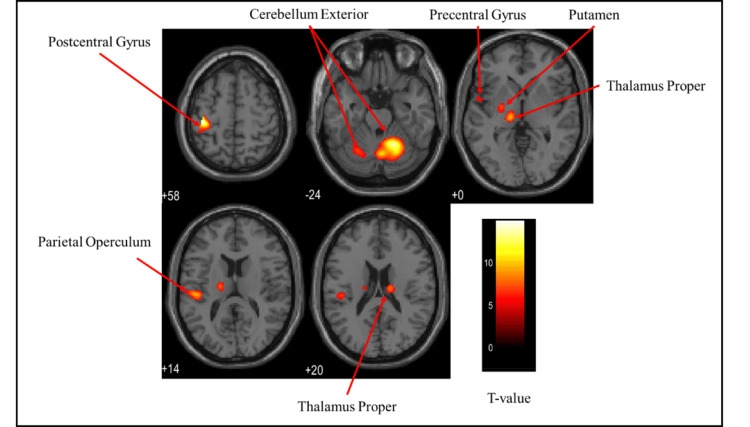
Brain activation regions in the female group during gripping of the medium stress ball The color bar represents T-values in the activation maps

**Figure 8:**
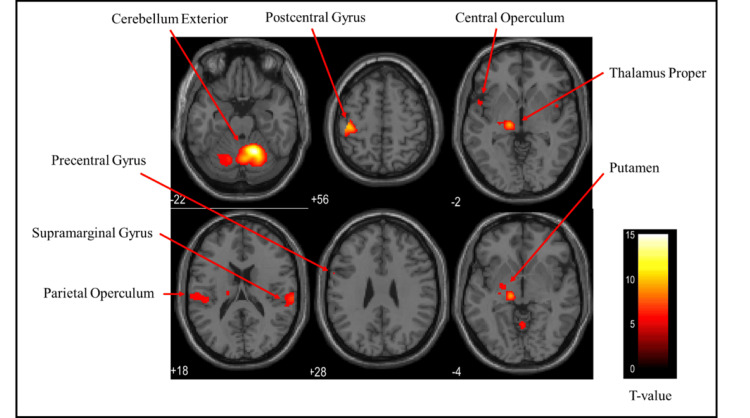
Brain activation regions in the female group during gripping of the hard stress ball The color bar represents T-values in the activation maps

Figures 6-8 and Table 2 present the brain regions activated in the female group during gripping of each type of stress ball (soft, medium, hard). Figure 6 shows activation areas during gripping of the soft stress ball, with significant activation in the cerebellum exterior, postcentral gyrus, thalamus proper, anterior insula, parietal operculum, putamen, and precentral gyrus. Figure 7 illustrates activation areas in the medium stress ball condition, revealing significant activation in the postcentral gyrus, cerebellum exterior, thalamus proper, parietal operculum, putamen, and precentral gyrus. Figure 8 depicts activation areas during gripping of the hard stress ball, with significant activation in the cerebellum exterior, postcentral gyrus, thalamus proper, central operculum, supramarginal gyrus, precentral gyrus, parietal operculum, and putamen.

**Table 2:** Brain activation regions and corresponding Montreal Neurological Institute (MNI) coordinates during gripping of each stress ball in the female group * One-sample t-test FWE: family-wise error

	Hemisphere	Locations	Cluster p－value (FWE)	Cluster size (voxels)	T－ value	X [mm]	Y [mm]	Z [mm]
Soft*	Right	Cerebellum Exterior	<0.001	2378	14.98	16	-54	-24
	Left	Postcentral Gyrus	<0.001	420	9.88	-40	-26	58
	Left	Thalamus Proper	<0.001	339	8.88	-14	-22	4
	Left	Anterior insula	<0.001	219	7.73	-48	0	2
	Left	Parietal Operculum	<0.001	166	7.54	-50	-24	16
	Left	Putamen	<0.001	128	6.95	-22	-6	2
	Left	Precentral Gyrus	0.010	3	5.84	-58	8	28
Medium*	Left	Postcentral Gyrus	<0.001	659	14.41	-38	-26	58
	Right	Cerebellum Exterior	<0.001	1713	13.14	18	-54	-24
	Left	Thalamus Proper	<0.001	515	9.47	-14	-22	4
	Left	Parietal Operculum	<0.001	189	9.09	-48	-24	14
	Left	Cerebellum Exterior	<0.001	206	8.73	-28	-62	-28
	Right	Thalamus Proper	<0.001	65	7.59	16	-16	20
	Left	Putamen	<0.001	72	7.10	-24	-8	2
	Left	Precentral Gyrus	0.027	12	5.47	-52	2	0
Hard*	Right	Cerebellum Exterior	<0.001	2479	15.09	18	-56	-22
	Left	Postcentral Gyrus	<0.001	454	10.12	-40	-26	56
	Left	Thalamus Proper	<0.001	363	9.66	-14	-20	-2
	Left	Central Operculum	<0.001	275	7.33	-48	0	4
	Right	Supramarginal Gyrus	<0.001	173	7.07	66	-24	18
	Right	Precentral Gyrus	<0.001	39	6.78	54	4	40
	Left	Parietal Operculum	<0.001	143	6.53	-52	-22	16
	Left	Putamen	0.006	28	6.00	-22	-10	-4
	Right	Central Operculum	0.013	51	5.73	48	2	10
	Left	Precentral Gyrus	0.036	1	5.34	-58	6	28

Brain activation regions in the male group

**Figure 9:**
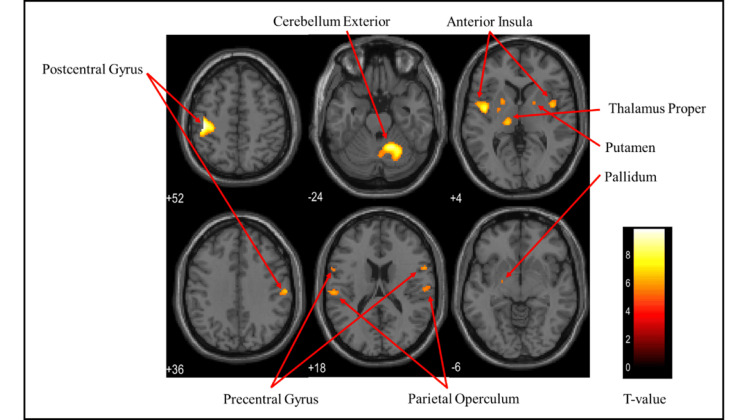
Brain activation regions in the male group during gripping of the soft stress ball The color bar represents T-values in the activation maps

**Figure 10:**
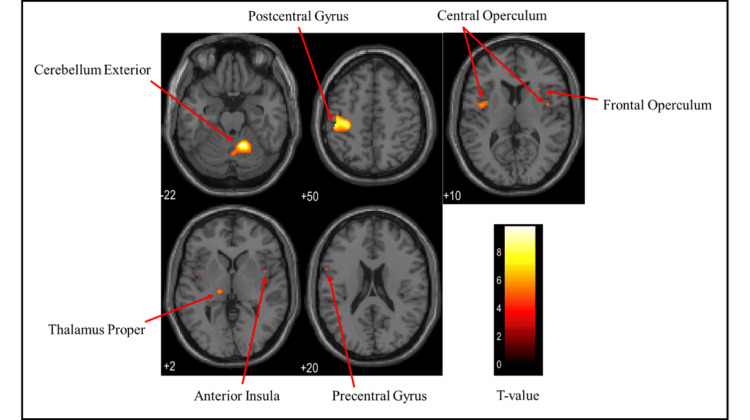
Brain activation regions in the male group during gripping of the medium stress ball The color bar represents T-values in the activation maps

**Figure 11:**
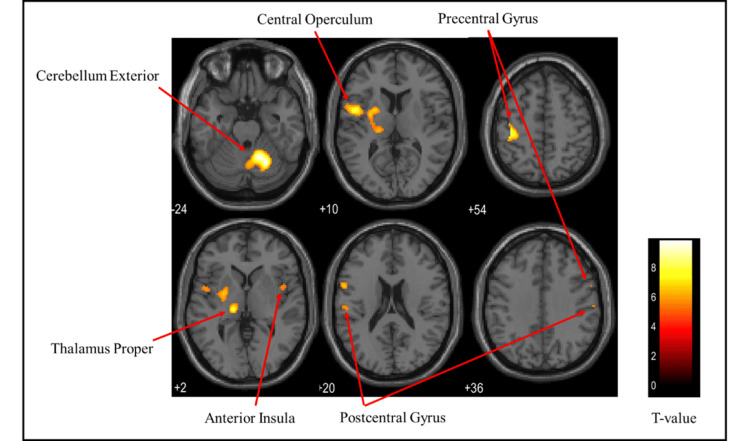
Brain activation regions in the male group during gripping of the hard stress ball The color bar represents T-values in the activation maps

Figures 9-11 and Table 3 present the brain regions activated in the male group during gripping of each type of stress ball (soft, medium, hard). Figure 9 shows activation areas in the soft stress ball condition, with significant activation in the postcentral gyrus, cerebellum exterior, anterior insula, thalamus proper, parietal operculum, pallidum, precentral gyrus, and putamen. Figure 10 illustrates activation areas in the medium stress ball condition, revealing significant activation in the cerebellum exterior, postcentral gyrus, central operculum, thalamus proper, anterior insula, frontal operculum, and precentral gyrus. Figure 11 depicts activation areas in the hard stress ball condition, showing significant activation in the cerebellum exterior, central operculum, precentral gyrus, thalamus proper, anterior insula, postcentral gyrus, and middle frontal gyrus.

**Table 3:** Brain activation regions and corresponding Montreal Neurological Institute (MNI) coordinates during gripping of each stress ball in the male group * One-sample t-test FWE: family-wise error

	Hemisphere	Locations	Cluster p－value (FWE)	Cluster size (voxels)	T－ value	X [mm]	Y [mm]	Z [mm]
Soft*	Left	Postcentral Gyrus	<0.001	633	9.94	-42	-26	52
	Right	Cerebellum Exterior	<0.001	814	9.11	16	-54	-24
	Left	Anterior insula	<0.001	244	8.16	-42	-4	4
	Left	Thalamus Proper	0.001	148	6.88	-20	-24	10
	Right	Anterior insula	0.001	122	6.76	42	0	4
	Right	Postcentral Gyrus	0.002	49	6.61	60	-18	36
	Left	Parietal Operculum	0.003	65	6.32	-56	-22	18
	Left	Pallidum	0.004	97	6.25	-20	0	-2
	Right	Precentral Gyrus	0.006	28	6.13	58	10	18
	Right	Putamen	0.007	18	6.07	20	4	2
	Right	Parietal Operculum	0.007	61	6.03	60	-20	22
	Left	Precentral Gyrus	0.017	22	5.70	-58	8	20
Medium*	Right	Cerebellum Exterior	<0.001	501	11.28	16	-54	-22
	Left	Postcentral Gyrus	<0.001	426	10.09	-32	-26	50
	Left	Central Operculum	<0.001	95	7.18	-40	-4	12
	Left	Thalamus Proper	0.003	23	6.49	-14	-22	2
	Right	Anterior insula	0.003	18	6.49	42	-2	12
	Right	Central Operculum	0.02	7	5.69	42	4	0
	Right	Frontal Operculum	0.022	7	5.65	32	18	10
	Left	Precentral Gyrus	0.028	9	5.56	-58	8	20
Hard*	Right	Cerebellum Exterior	<0.001	3930	16.4	18	-56	-22
	Left	Central Operculum	<0.001	365	8.35	-46	2	10
	Left	Precentral Gyrus	<0.001	338	8.33	-38	-24	54
	Left	Thalamus Proper	<0.001	596	7.98	-16	-22	2
	Right	Anterior insula	0.008	37	6.03	44	4	4
	Left	Postcentral Gyrus	0.013	14	5.86	-54	-20	20
	Right	Precentral Gyrus	0.014	3	5.82	56	8	38
	Right	Postcentral Gyrus	0.022	6	5.65	62	-18	36
	Right	Middle Frontal Gyrus	0.041	1	5.40	54	6	42

Contrast results of differences between male and female groups

**Figure 12:**
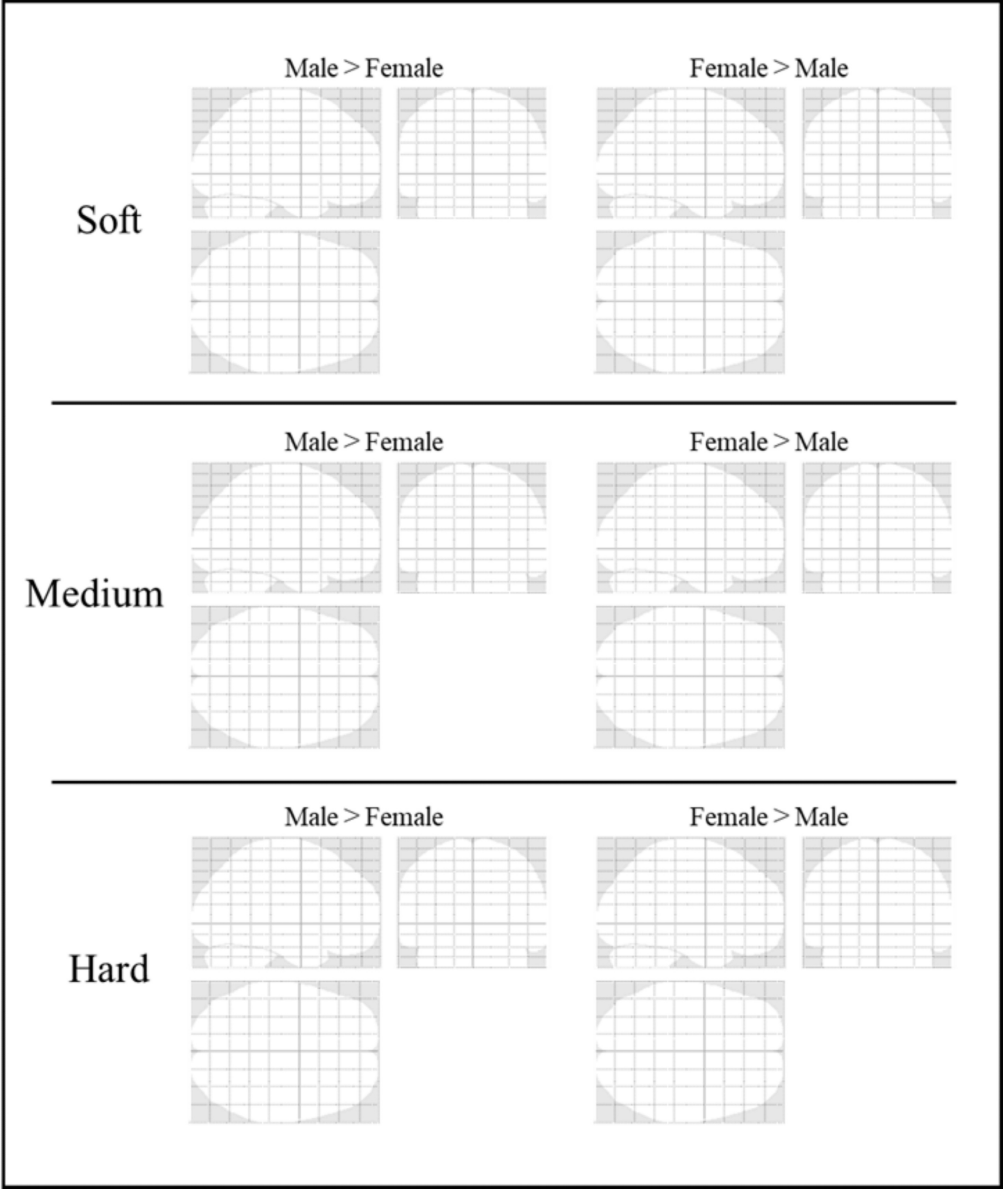
Glass brain images showing contrasts between male and female groups during gripping of each stress ball (soft, medium, hard) The figure displays the difference maps in three planes: axial, sagittal, and coronal

Figure 12 presents glass brain images illustrating contrasts between male and female groups during gripping of each type of stress ball (soft, medium, hard). The glass brain images provide an overview of the spatial distribution of activation regions. In this analysis, no significant differences in brain activation were observed between males and females for any of the stress ball conditions.

Questionnaire results

Table 4 presents comfort scores when gripping each stress ball, comparing male and female groups. For the soft stress ball, the mean comfort score was 7.2 ± 1.8 for males and 6.7 ± 3.7 for females, with no significant difference (p = 0.143). For the medium stress ball, males reported a significantly higher comfort score (6.8 ± 1.5) than females (5.9 ± 3.9) (p = 0.029). For the hard stress ball, the mean scores were 5.4 ± 1.6 for males and 4.6 ± 3.1 for females, with no significant difference (p = 0.053).

**Table 4:** Comfort scores during gripping of stress balls in male and female groups Higher scores indicate greater comfort, lower scores indicate discomfort, and a score of 5 represents a neutral response. * Significant difference－Mann-Whitney U test ns－Not significant difference－Mann-Whitney U test

	Male		Female		U	Z	p
	Mean	SD	Mean	SD			
Soft	7.2	1.8	6.7	3.7	926.5	1.465	0.143^ns^
Medium	6.8	1.5	5.9	3.9	998.5	2.189	0.029^*^
Hard	5.4	1.6	4.6	3.1	974.5	1.934	0.053^ns^

Table 5 shows perceived hardness scores for each stress ball in males and females. For the soft stress ball, males rated it significantly softer (2.4 ± 1.3) than females (3.1 ± 5.0) (p = 0.041). For the medium stress ball, the mean hardness scores were 4.7 ± 1.7 for males and 5.2 ± 5.0 for females, with no significant difference (p = 0.196). For the hard stress ball, males scored 6.7 ± 1.6 and females 7.1 ± 4.8, with no significant difference (p = 0.226).

**Table 5:** Hardness scores during gripping of stress balls in male and female groups Higher scores indicate greater perceived hardness, lower scores indicate greater perceived softness, and a score of 5 represents a neutral response. * Significant difference－Mann-Whitney U test ns－Not significant difference－Mann-Whitney U test

	Male		Female		U	Z	p
	Mean	SD	Mean	SD			
Soft	2.4	1.3	3.1	5.0	579	-2.046	0.041*
Medium	4.7	1.7	5.2	5.0	651	-1.293	0.196^ns^
Hard	6.7	1.6	7.1	4.8	659.5	-1.211	0.226^ns^

## Discussion

In this study, we used fMRI to measure brain activity in 77 young adults during an active tactile stimulation task involving stress balls of varying hardness, examining the relationships among stimulus intensity, sex, and subjective evaluations. Brain regions primarily associated with sensorimotor functions, including the postcentral gyrus, precentral gyrus, thalamus proper, and cerebellum, were consistently activated across all participants. These findings suggest that the fundamental neural substrates for sensory and motor processing during active grasping movements are stably engaged regardless of sex. This indicates that brain responses to tactile stimulation are largely similar between males and females.

Differences in brain activation patterns according to hardness

In this study, robust activation in the postcentral gyrus, precentral gyrus, thalamus proper, and cerebellum was consistently observed during active grasping tasks with stress balls of different hardness levels (soft, medium, hard). These regions play central roles in sensorimotor processing and have been consistently reported in tactile stimulation and voluntary motor tasks [6,17-19]. The thalamus functions as a relay station for sensory input, while the primary somatosensory and motor cortices serve as the main regions for sensory reception and motor output, respectively. The cerebellum is involved in motor coordination and feedback regulation, and recent studies have also implicated it in sensory processing and predictive control [20], highlighting its functional diversity in line with the present findings.

A noteworthy result was the activation of higher-order cognitive and affective regions, including the angular gyrus, posterior cingulate gyrus, and hippocampus, observed specifically when participants gripped the medium stress ball. The angular gyrus is thought to integrate extrinsic connectivity and intrinsic regional properties shared with other association cortex areas [21,22]. It is linked to self-referential processing and internal attention, and, together with the posterior cingulate gyrus, forms part of the default mode network which is associated with conscious introspection and emotional evaluation [23]. The hippocampus, beyond its role in memory, is involved in emotional value judgment and spatial imagery processing [24,25]. The medium stress ball was rated as providing moderate stimulation, likely eliciting evaluative processing of comfort/discomfort and engaging higher-level processes that assign meaning to bodily sensations. This may reflect the “Goldilocks effect,” where the sensory stimulus is neither too strong nor too weak [26].

In contrast, activation during the hard stress ball condition was mainly confined to motor-related regions, with limited engagement of higher-order cognitive areas such as the frontal lobe and cingulate cortex. This suggests that strong grasping stimuli were processed primarily as task load without substantial emotional or evaluative processing. Previous reports have shown that excessive stimulation can suppress the involvement of affective systems [27], and the present results support this interpretation.

Differences in brain activation patterns by sex

This study examined sex differences in overall brain activation patterns. In the female group, activation was observed in regions associated with sensory integration and emotional processing, such as the supramarginal gyrus and parietal operculum. In contrast, the male group showed activation in prefrontal regions linked to cognitive control, including the frontal operculum and middle frontal gyrus, as well as regions associated with subjective emotional experience and affective processing, such as the insula. However, contrast analyses revealed no statistically significant differences in brain activation between males and females. This lack of difference may be related to the nature of the active tactile stimulation used in this study, which was simple and predictable (e.g., 5 kg force applied once per second) and may not have been sufficiently complex or intense to elicit substantial emotional changes. These findings align with previous research reporting similar sensitivity levels to simple stimuli across sexes. For example, Lillqvist et al. found no sex differences in sensitivity to simple olfactory stimuli [28], and studies using simple hand-cue stimuli have also reported no sex differences in judgment or sensitivity [29].

To our knowledge, this study is the first to use fMRI to investigate sex differences in sensitivity to active tactile stimulation and found no sex differences in brain activation regions under simple active stimulation conditions. Nevertheless, although no statistically significant sex differences were detected in fMRI contrast analyses, subjective evaluations indicated that males reported significantly higher comfort scores than females, specifically under the medium condition. This suggests that, although clear sex differences in neural activity were not observed, tendencies related to subjective sensitivity and evaluative processing may differ between sexes. Considering that males showed activation in cognitive control areas such as the frontal operculum and prefrontal cortex during the medium condition, it is possible that males cognitively perceived this condition as a manageable or comfortable task load. Furthermore, the exclusive activation of the insula in males during the medium condition suggests that males may have engaged in more refined emotional and internal evaluations of the stimulus under this condition [30].

No significant differences in brain activation regions were observed between sexes in the contrast analyses for any of the soft, medium, or hard conditions. The glass brain images also revealed no substantial differences in overall activation distribution patterns, supporting the presence of common neural substrates across sexes. Many studies on sex differences have reported variations in tasks involving emotion, empathy, and reward processing, including social judgment [31-33]. However, such differences are less likely to emerge in physical sensorimotor tasks such as the one used here. Additionally, the relatively large sample size of 77 participants in this fMRI study, with no statistically detected sex differences, suggests that any sex effects are small or may be overshadowed by individual variability. Factors such as subjective perception and grip strength during grasping tasks may influence brain activity. Future research should address not only sex-related effects but also individual characteristics such as grip strength, sensory thresholds, and emotional tendencies.

Limitations

This study aimed to elucidate brain activation patterns in response to active tactile stimulation and potential sex differences, yielding valuable findings; however, some limitations should be considered. First, the study population was limited to healthy young adults aged 20 years and older (n = 77) and did not account for age-related changes in neuroplasticity or tactile sensitivity. Previous studies have reported that cognitive processing of tactile stimuli changes with aging [34-36]; thus, it remains to be determined whether these findings generalize to older adults or populations at different developmental stages.

Second, the tactile stimuli in this study varied only in hardness, without including other features such as vibration, temperature, or texture. As real-life tactile experiences are multidimensional, future research using more complex and naturalistic stimuli is warranted.

Third, while fMRI offers high spatial resolution, its temporal resolution is limited, making it less suited to capturing dynamic processes such as immediate emotional responses or shifts in attention to tactile stimuli. Additionally, there may be temporal discrepancies between brain activity and subjective evaluations of comfort or discomfort. Therefore, complementary assessments incorporating simultaneous psychophysiological measures, such as skin conductance responses and heart rate variability, are recommended for future studies.

## Conclusions

To our knowledge, this study is the first to elucidate brain responses to active tactile stimulation from both neurological and subjective perspectives, focusing on stimulus intensity and sex differences. Consistent activation of sensorimotor regions was observed across all hardness conditions, suggesting that a stable neural basis for basic tactile processing exists regardless of sex. Although statistical analyses of fMRI data did not reveal clear sex differences in brain activation patterns, males reported higher comfort than females only in the medium condition during subjective evaluations. This indicates that even when neural activity is comparable, sex differences may emerge in sensory evaluation processes. Therefore, future research should aim to capture more precisely how sex and individual characteristics influence subjective evaluations and behavior. Further studies incorporating age, individual variability, and a wider range of tactile stimuli are warranted.
